# What We Seem and What We Are

**Published:** 1880-01

**Authors:** Ausburn Towner


### WHAT WE SEEM AND WHAT WE ARE.

AUSBURN TOWNER.

I have known instances, as has doubtless every reader of the BISTOURY, where a youth of extremely humble parentage has quitted the scene of his birthplace and has gone into other lands. He succeeds in his undertakings, becomes rich and famous, probably marries into a fine family and loses most, if not all of the characteristics that mark those of his own flesh and blood whom he has left behind. It is not a very manly or generous feeling, but there have been instances where such a youth has grown to be ashamed of his origin and has almost denied his father and the mother who bore him. Now it is very much the same way with the two races that are largely represented in this country and that certainly form the most active and thriving portion of it. Think a moment. In religion, we are Jews, that is, worshipping a Jew as a God ; in the abstract sciences, the arts and literature we are Greeks ; in experimental science, and natural, physical, mechanical and practical knowledge, we are Arabs ; in the ways of politics and government, we are Romans, and in country we are Indians. Yet we surely are neither Jews, Greeks, Arabs, Romans or Indians. We are very much like the youth spoken of at first, forgetful, if not ashamed of the blood that flows in our veins and of those ancient nations among whom were our ancestors. Can it be that they are entirely unworthy our consideration and attention ; that these people from whom have sprung the men who are in the advance guard of civilization, have nothing that merits our attention in comparison with the other nations their contemporaries, about whom we hear so much ? I prefer to believe it is not so ; and for myself, I would at any rate rather have, and would wear closer to my heart, a simple memorial of my own father's life, humble and unpretentions as it may have been, and won in the midst of opposing surroundings, than the richest memorial, the stateliest grandeur that belongs to some other family. I would set more store by the anvil and hammer the lapstone and last of him or those in whose veins flows the same blood that now gives me life, than in all the armorial bearings and heraldic devices that mark the origin of the most noble family, who are none of mine. And at the same time, if these *were* mine, so much the more pride would I take in them.

But there is everything in those old nations, the Saxons and Celts, from which we are descended, in which we can take a just pride and can claim for them a position not at all inferior in most respects to those peoples who are called classical.

I would not institute any comparison between our ancient forefathers and their neighbors, for it would be needless. I would simply and briefly show that not all that we have and are with the exception of our bodies, come to us from other races than our own ; that our ancient fathers and mothers besides our breath and blood, bequeathed to us that which is of as much interest and value as what may have come to us from other sources.

As to the prevalent religion, we have the same tree that our fathers had ; that which is now observed, is only a graft upon the parent trunk. The Druids or men of the Oaks, worshipped and were priests of One God and they taught the immortality of the soul, even while the cultured Greeks and Romans had a pantheon and a mythology that numbered tens of thousands, and were dimly guessing at the hereafter. The observances of these Druids still live in the season now before us. To them, the mistletoe and the holly were objects of great veneration and were gathered with ceremonies of peculiar significance. The sacred character of these plants has passed away to be sure, but there still lingers around them, a romantic and fanciful notion that has drifted down to us through these many long years, and will probably never be entirely obliterated. Easter also is an ancient Druidical festival, in all probability counting its origin ages before Christ arose. It retains its name and occurs in the same season as of old, but its significance has changed.

It is almost trite to recall to the mind of the reader, that he has every day on his lips, the name of one or another of the inferior deities highly regarded by our ancestors. Tuesday is called for Tuisco the Saxon God of justice and peace ; Wednesday from Wodin the heavenly Secretary of War ; Thursday from Thor, the representative of strength and manly beauty ; Friday from Freia, the goddess of Love.

As an offshoot of this religions sentiment, we have left to us, the suggestion, at least, of the Gothic architecture, which for church purposes is esteemed the most apt and suitable. The worship of our ancestors was all performed in the open air, the most magnificent forest being selected and consecrated for that purpose. I know of no more suitable place in which to worship God than in the midst of an architecture of his own fashioning, and I shrewdly suspect that our friends who annually betake themselves to the woods and make the whole air vocal and melodious with their prayers and hymns are but following out the instincts of their ancient forefathers.

Still further, springing from the ancient religion, all of the thousand and one superstitions especially those relating to our home life and our personal concerns, the neglect of which at the moment gives us a sting of apprehension, and the memory of which quickly passes away; such as the disastrous consequences attending upon seeing a new moon the first time over the left shoulder ; the fear of going back for anything you may have forgotten after you are once started ; putting on a stocking or an under-garment wrong side out ; hanging a horseshoe over a doorway for good luck ; the mysterious connection between a curious little bone that lies close to the breast of a fowl and one's wishes ; the strange shrinking fate of pork, when it is cooked, if it has been killed during the waning of the moon ; these and many others which the reader may be able to recall readily and which are very close to our hearts, think as we may and reason as we may, though they are childish and without reason, were subjects of pious concern and thoughtfulness, in the early days of that people of whom we are the lineal descendants. And the multitude of proverbs, those sayings which have been called the "wisdom of many and the wit of one," which enrich our daily speech and literature, giving a pith and point to a sentiment or rounding off a sentence like a quick stroke from a keen blade, can mostly be traced backward to those thoughtful and accurately observing men of the oaks, and to the times long before writing was known, or practiced ; when speech was apt to be curt and sharp, and men used fewer words than they do now to express their ideas.

Common observances too which we see almost every day of our lives, and for which we can give no reason that is satisfying to ourselves had their origin in these times of which I speak. We comply with them unquestioningly, because our Fathers did. There are All Fools Day, Harvest Home, All Halloween, the May Day Festival, and as an apt and easily perceived illustration, why are bonfires lit in times of glorification or victory ? They fill the air with smoke and cinders, distress the police force, keep the watchman at the fire tower unusually vigilant, and leave a great black heap of cinders for the street commissioner to cart grumblingly away the next morning. What possible connection can there be between jubilant feelings and a great roaring dangerous mass of flame, with its numerous tongues lapping up the air, or between exuberant happiness and the destruction by fire, of that which could be put to far more useful purposes ? Ah ! there is where our blood comes in. Any analogy drawn from anything modern could only be exceedingly fanciful, and would be just as untruthful. We build bonfires because our ancient forefathers did. The fact remains ; the reason for it is gone. They did it as an act of worship, glorifying themselves and their great sun God, thinking in this manner, and by imitating even rudely the source of light, and heat, and strength, they were giving thanks to him for the benefits which he had conferred upon them, and worshipping him in a manner that he could the best understand and appreciate.

Lying close to the religious sentiments of our ancestors, is that belief, which has no parallel among any of the other nations of the world, that each of the four elements were tenanted by sprites or miniature mites of beings, gnomes in the earth, salamanders in the fire, elves in the air, and sprites in the water. This fancy was a living and breathing reality to those ancient people, and they live in our days in the realm of childhood, making it, with its fairies, its Puck, Tom Thumb, Jack the Giant Killer and Santa Claus, who is but a magnified elf at the best, a land and kingdom all by itself. The fancy has done more than this too. It has given to our literature, some of the most exquisite creations that have adorned its pages. Who can deny its mythical beauty who has heard of the legends still current and believed in Ireland, of the little folks dancing in the meadows under the moonlight ; of the enticing Will o' the wisp, who beguiles the belated and deceived traveller into swamps and dangerous places, by the wandering radiance of his lantern ; of the

"Airy voices who syllable men's names
"On sands and shores and desert wilderness ;"

who has read of Shakespeare's Ariel ; who knows the delicious story of Undine ; who has read Robert Burns's Halloween, or Tam O'Shanter, or that most exquisite creation of the imagination : "The Culprit Fay," by Rodman Drake ?

There were a class of men among our ancestors unlike any of any other race or nation, called Bards. The position of Poet Laureate, now held by Alfred Tennyson, is lineally descended from these singers. They were attached to royal or noble houses, and it was their duty to sing the deeds of valor that they had witnessed, or of which they had been told, mingling them with stories of love and romance. Some of their productions that have come down to us we need not be ashamed to compare with those of Homer or any of the other much vaunted classical writers. The works of these Bards were the origin and model of the modern novel. If you would read something that is tinctured with the spirit of the poetry of these Bards, more perhaps in matter than in manner of construction, there are Longfellow's "Tales of a Wayside Inn," and the "The Skeleton in Armor ;" Walter Scott's longer poems, especially that one entitled "The Lay of the Last Minstrel," in which, as he himself says, he hopes to have caught somewhat the refinement of modern poetry, without losing the simplicity of his original model. And besides these, there are Tennyson's "Idyls of the King."

One distinction our ancestors enjoyed placing them so far in advance of other contemporary nations that we may regard them with just pride on its account : their deference to, and respect for woman. While in other lands she was condemned to the most abject inferiority either as a drudge or a toy, among our ancestors, women were held as equal in all respects, in some, superior to men. Women are much as men make them, and while the one way, giving us the worthy as exceptions, has produced such conspicuous examples of evil as Semiramis, Cleopatra, Aspasia, the Borgias, and a score or more of others that blot the pages of the Eastern chronicles, the other way giving us the bad as exceptions, can show those noble women, the mother of Attila, Brunhilda, Boadicea, Elfrida, and many more like them that brighten the history of our ancestors.

We are indebted to our Saxon ancestors for what is popularly called one of the bulwarks of liberty—the trial by jury. It has its counterpart in the jurisprudence of no other race on the face of the globe. Those representative bodies that are the distinguishing features of the government of our country too, our Congress in the National government, and our Assemblys and Senates in the States, had their origin in the Saxon Wittenagemots, or the periodical assemblages of the wise men among our ancestors. In another direction of human endeavor, we are indebted to the Celtic crowth, or rote, for that noblest and most perfect of musical instruments. the modern violin. Our knowledge of shipping and navigation we owe entirely to our Saxon ancestors, this being demonstrated by the fact that every block, yard, sheet or pin, from keel to topmast of a vessel, is known to our sailors now and called by them in the same words that were given to them centuries ago by our restless, roving ancestors.

The BISTOURY itself would hardly suffice to set forth, even to a limited extent, how much we really owe to our own fathers and mothers, although until within a very recent period the subject has been kept rather sedulously in the background. Those who have looked into it and have discovered what a field there is for exploration, will be glad to know that in one or two of our colleges there has been established a professorship of Anglo-Saxon literature and history. It is about time. One of the reasons often given for the study of the dead, or what are called the classical languages, is, that by becoming acquainted with them we will the more thoroughly understand our own tongue. That has always seemed strange to me, especially as such little time is bestowed on the study of our mother-tongue itself. And yet, unadulterated by the mixture of foreign words, the language given to us by our Anglo-Saxon forefathers is the strongest, sweetest, purest tongue ever spoken by man. It is the best for love, the best for hate, the best for eloquence and poetry, the best for manly sentiments, and for the talk of a mother to her babe. It has words that have no synonyms in other languages. Take that one word —home. No other nation has a word, or set of words, that carry so much significance with it as does it. Had our ancestors bequeathed to us nothing but the language that we speak, they would be entitled to more consideration than they get from us now.

I do not wish to be considered as setting a lower value upon what appertains to those things that belong to other races and nations, but I would set a higher value upon what belongs to us by right of blood. Give to these the first place, and to those, if you choose, the second. It is not necessary to forget Alexander the Great, and Cæsar, Sophocles and Solon, even if we do hear a little more about the Venerable Bede, King Arthur, Tallesin of the radiant face, Merlin, and Virgil the Bishop of Salisbury.

